# Investigation of photoacoustic tomography reconstruction with a limited view from linear array

**DOI:** 10.1117/1.JBO.26.9.096009

**Published:** 2021-09-28

**Authors:** Min Ai, Jiayi Cheng, Davood Karimi, Septimiu E. Salcudean, Robert Rohling, Purang Abolmaesumi, Shuo Tang

**Affiliations:** a University of British Columbia, Department of Electrical and Computer Engineering, Vancouver, Canada; b University of British Columbia, Department of Mechanical Engineering, Vancouver, Canada

**Keywords:** photoacoustic tomography, linear array transducer, iterative reconstruction

## Abstract

**Significance:** As linear array transducers are widely used in clinical ultrasound imaging, photoacoustic tomography (PAT) with linear arrays is similarly suitable for clinical applications. However, due to the limited-view problem, a linear array has limited performance and leads to artifacts and blurring, which has hindered its broader application. There is a need to address the limited-view problem in PAT imaging with linear arrays.

**Aim:** We investigate potential approaches for improving PAT reconstruction from linear array, by optimizing the detection geometry and implementing iterative reconstruction.

**Approach:** PAT imaging with a single-array, dual-probe configurations in parallel-shape and L-shape, and square-shape configuration are compared in simulations and phantom experiments. An iterative model-based algorithm based on the variance-reduced stochastic gradient descent (VR-SGD) method is implemented. The optimum configuration found in simulation is validated on phantom experiments.

**Results:** PAT imaging with dual-probe detection and VR-SGD algorithm is found to improve the limited-view problem compared to a single probe and provide comparable performance as full-view geometry in simulation. This configuration is validated in experiments where more complete structure is obtained with reduced artifacts compared with a single array.

**Conclusions:** PAT with dual-probe detection and iterative reconstruction is a promising solution to the limited-view problem of linear arrays.

## 1 Introduction

Photoacoustic (PA) imaging has many potential applications in biomedical imaging due to its unique properties such as deep penetration and biochemically specific contrast from hemoglobin.^1^ Using a linear array transducer for PA imaging has the advantage of wide availability, low cost, and compatibility with existing clinical ultrasound (US) imaging. PA imaging systems based on linear array transducers have been studied for various applications such as breast cancer,^2^^,^^3^ prostate cancer,^4^^–^^6^ and thyroid cancer.^7^ Neuschler et al.^2^^,^^3^ investigated PA imaging to diagnose benign and malignant breast masses in patients. Their PA imaging device contained a handheld linear transducer of 128 elements (4 to 16 MHz at 20 dB power point). PA imaging enabled the visualization of radiating vessels in the peripheral zone and multiple boundary zone vessels, which were significant features in distinguishing benign from malignant breast tissues. Their study showed that combining PA imaging with US imaging provided 14.9% higher diagnostic specificity than grayscale US alone. Horiguchi et al.^4^^,^^5^ developed a PA imaging system equipped with a conventional linear probe (linear array, 128 elements, 8 MHz central frequency) and a transrectal probe (convex array, 128 elements, 6 MHz central frequency), respectively, for imaging prostate. The linear probe was applied to *ex vivo* imaging of resected prostate specimens, whereas the transrectal probe was applied to intraoperative *in vivo* patient imaging. In their pilot study, they have shown that PA imaging was able to locate microvascular complex in the neurovascular bundle^4^ and provide information about microvascularity such as density, total vascular area, and length, which correlated with prostate cancer.^5^ However, PA imaging based on linear array transducer has a limited-view problem, where the PA signal is only received from a limited detection angle, as restricted by the dimension of the linear transducer. The limited-view detection results in an ill-posed reconstruction and leads to artifacts and blurring.^6^ In those clinical studies mentioned above, although the PA images added complementary information to the conventional US images, PA imaging with linear array transducer was not able to obtain clear structural image but only showed regions with high PA contrast. To localize the PA contrast, the PA image was overlaid on top of a co-registered US image that provided the anatomical structure. Therefore, the limited-view problem has hindered the potential of PA imaging based on linear array transducer for broader clinical applications.

To address the limited-view problem, current methods focus either on hardware improvement or software reconstruction. Hardware improvement usually relates to the change of detection geometries. Li et al.^7^ achieved full-view detection with a linear array transducer by rotating the sample at 360 deg. Their system was only able to image samples with a diameter <20  mm. Yang et al.^8^ achieved full-view detection by circularly rotating a linear array transducer around the animal. Their *in vivo* experiment on rat brain imaging showed clear network of blood vessels. However, rotating the sample or the linear array transducer may not be applicable to imaging patients. Huang et al.^9^ placed an acoustic reflector at 45 deg angle to reflect the PA wave propagating in other direction back to a linear array transducer, equivalent to adding a virtual array perpendicular to the real one to double the detection view. However, it can be challenging to put a reflector in clinical imaging of patients and the acoustic attenuation is much higher for the reflected PA wave due to its longer pathlength.

Previously, we have demonstrated an approach of using dual-linear array transducers to double the detection angle, which significantly improved the image quality.^10^ The two linear array transducers were positioned to fit the anatomical geometry of the imaging site, and a calibration method was developed to calibrate the relative positions of the two transducers by transmitting US signal from one probe to the other.^10^ This method allowed the positioning of two linear array transducers in a flexible angle to improve the detection view. Dual-linear array transducers were also utilized by Zhang et al.^11^ in their x-ray-induced radiation-acoustic and US imaging. The image quality was significantly enhanced by merging the individual images obtained from the two transducers, respectively. Therefore, a dual-probe configuration can address the limited-view problem and thus greatly improve the quality of PA imaging based on linear array transducers.

PA image reconstruction by conventional delay and sum (DAS) is restricted by limited-view detection and results in artifacts and blurring. Iterative model-based reconstructions have been reported to reduce artifacts and improve the PA image reconstruction. Most iterative reconstructions were studied for full-view detection geometry, where the samples were enclosed by the transducer elements. Only a few papers dealt with linear-array transducers. Guo et al.^12^ applied iterative reconstruction of PA imaging with a linear array transducer, where their process contained a non-linear conjugate gradient descent algorithm with total variance (TV) regularization. They investigated the under-sampling issue when fewer numbers of detection elements were used to accelerate the data acquisition (DAQ) speed. Shang et al.^13^ developed an iterative approach for PA imaging with a linear array transducer, where they built a forward model based on the actual measurement of impulse response at each point on the image plane. However, the measurement of impulse response was not feasible for clinical imaging dealing with different patients and their approach required long processing time. Variance-reduced stochastic gradient descent (VR-SGD) is an upgraded gradient descent algorithm that can achieve less computational time than full gradient descent (FGD) algorithm and faster convergence than stochastic gradient descent (SGD) algorithm.^14^^,^^15^ VR-SGD has been applied to computed tomography image reconstruction and proved to be simple and effective.^16^ VR-SGD has not been implemented in PA imaging before.

In this paper, we will investigate PA image reconstruction under the limited-view condition that commonly exists in systems using linear array transducers. Through simulation, we investigate and compare various detection geometries enabled by linear arrays, including traditional single array, a four-probe square-shaped configuration mimicking full-view detection, and dual-probe configuration that are promising for increasing the limited view. In addition, model-based VR-SGD algorithm will also be implemented for PA imaging to further address the limited-view problem and compare to traditional DAS. As guided by the simulation results, the promising detection geometry of dual-probe configuration and VR-SGD algorithm will further be implemented and validated in experiments on phantom studies. Our study shows that the proposed approach is feasible and can effectively address the limited-view problem, which can inspire more potentials of utilizing linear array transducers in PA imaging for clinical applications.

## 2 Method

### 2.1 VR-SGD Algorithm

In a model-based iterative reconstruction, a forward operator projects a received PA wave from an estimated initial pressure distribution. The iterative reconstruction attempts to minimize a cost function between the measured PA signal and the projected PA wave. A regularizer related to signal sparsity is usually added to the cost function to control over-fitting. Minimization of the cost function can be solved by various optimization methods such as gradient descent algorithms.

**Fig. 1:**
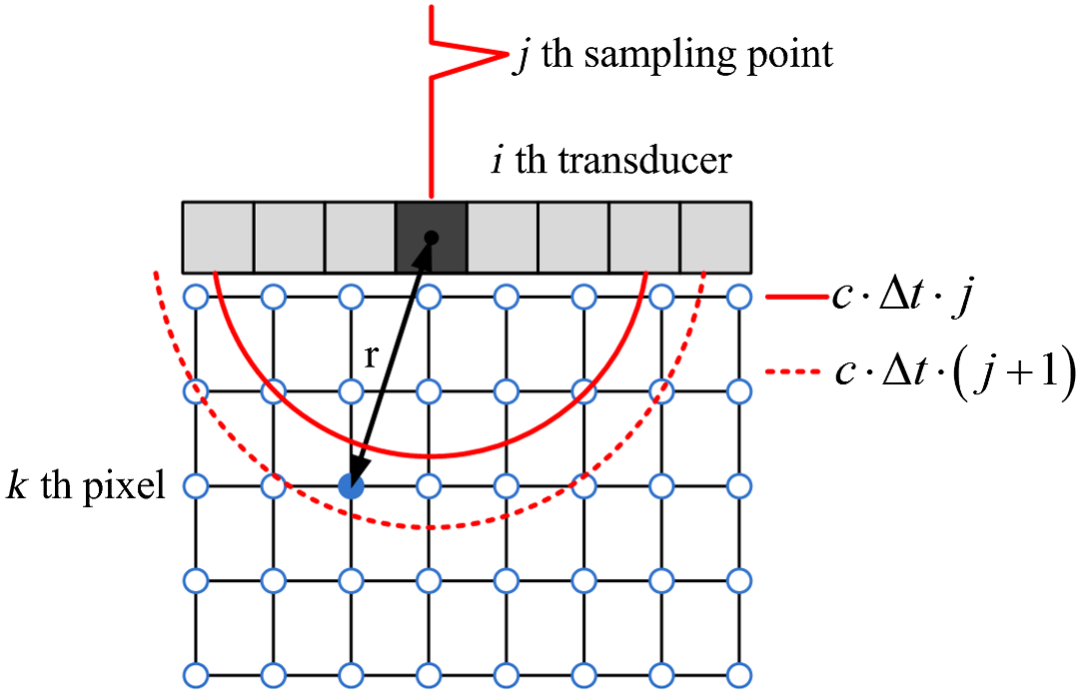
Illustration of the generation of the projection matrix.

In matrix format, a discrete forward model can be used to map the initial pressure distribution to the received PA data. The discrete model has been reported in literature and will only be explained briefly here.^12^^,^^13^^,^^15^ For a linear array transducer, the PA data received by each transducer element can be expressed as

$$G_{i}=A_{i}X,\;i\in [1,Q],$$ (1)

where X represents the initial pressure distribution, $G_{i}$ represents the acoustic velocity potential received by the i’th transducer element, $A_{i}$ is the submatrix that maps X to $G_{i}$, and Q is the total number of transducer elements. For matrix operation, X and $G_{i}$ are vectorized. For a 2D image of n×n pixels, X is vectorized into an $n^{2}\times 1$ vector. The vectorized $G_{i}$ has a size of T×1, where T is the number of temporal sampling points. The submatrix $A_{i}$ has the size of $T\times n^{2}$. Figure 1 illustrates the generation of the projection matrix, where i, j, and k are the indices for the transducer element, temporal sampling point of the PA data, and pixel position on the image, respectively. Here r is the distance from the i’th transducer element to the k’th pixel, Δt is the temporal sampling period, and c is the speed of the pressure wave.

The entries of the submatrix $A_{i}$ can be built by the interpolating-based discrete to discrete photoacoustic tomography (PAT) model as^15^

$$A_{i}(j,k)=\{\begin{array}{ll} \frac{\left(1-|\frac{r}{c\cdot \Delta t}-j|)\cdot D(i,k\right)}{2\pi r}, & \text{when }|\frac{r}{c\cdot \Delta t}-j|<1\\ 0 & \text{else} \end{array},\;i\in [1,Q],\;j\in [1,T],\;k\in [1,n^{2}].$$ (2)

For any pair of the i’th transducer element and k’th pixel on the image, we can find their distance r. Since r/(c·Δt) is generally not an integer, only those neighboring integers j that satisfy $|\frac{r}{c\cdot \Delta t}-j|<1$, meaning their distance is within one sampling point, the value of the matrix entry $A_{i}(j,k)$ is non-zero and a smaller value of distance $|\frac{r}{c\cdot \Delta t}-j|<1$ leads to a larger entry value in $A_{i}(j,k)$ and vice versa. In Eq. (2), a directivity weighting factor D(i,k) is incorporated into the discrete forward model, which is derived from the angle subtended from the k’th pixel on the image to the i’th transducer element. The factor 1/2πr describes the pressure wave decay due to geometrical spreading in 2D PAT imaging.

By considering all the transducer elements, a full projection map X↦G can be expressed as

$$G=[\begin{array}{l} G_{1}\\ G_{2}\\ \vdots \\ G_{Q} \end{array}]=[\begin{array}{l} A_{1}X\\ A_{2}X\\ \vdots \\ A_{Q}X \end{array}]=AX,\;\text{where }A=[\begin{array}{l} A_{1}\\ A_{2}\\ \vdots \\ A_{Q} \end{array}].$$ (3)

The full projection matrix A has a size of $Q\times T\times n^{2}$ that is usually very big. For example, for an image of 128×128  pixels, and a linear array transducer of 128 elements and 1000 temporal sampling points, the full projection matrix A contains $2.1\times 10^{9}$ entries, whereas the submatrix $A_{i}$ only contains $1.6\times 10^{7}$ entries.

Optimization is carried out by solving a least-squares cost function that contains a misfit term and a regularization term:

$$J(X)=\frac{1}{2}{\left\|AX-G\right\|}_{2}^{2}+\lambda TV(X),$$ (4)

where ${\left\|\cdot \right\|}_{2}$ is the entry-wise L2-norm. Normally, the gradient matrix of the initial pressure map is sparse. Thus a TV term is used for regularization to avoid over-fitting and λ is the tuning parameter. The iteration processes an estimated image X to minimize the cost function.

**Fig. 2:**
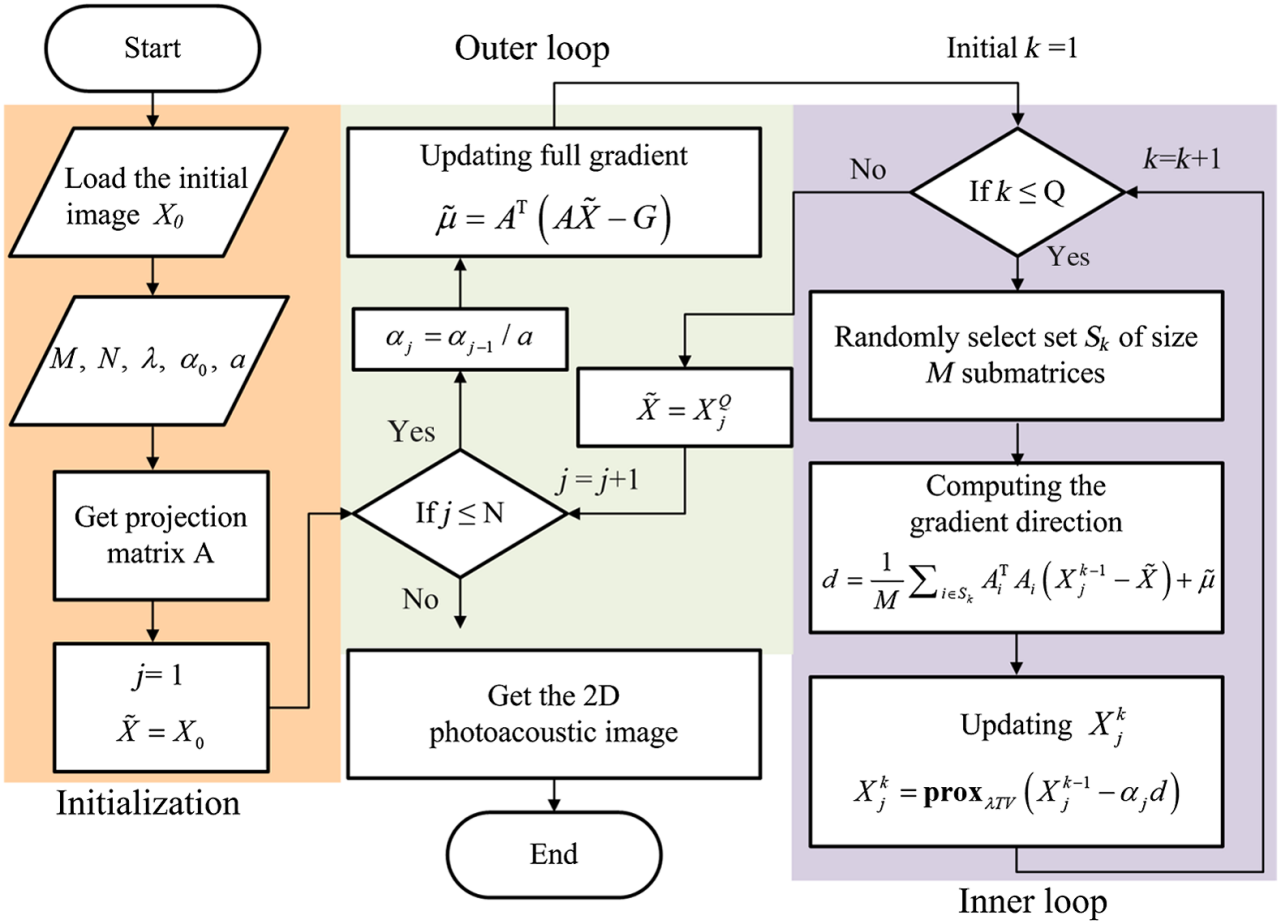
Flowchart of VR-SGD algorithm for PAT reconstruction.

The flowchart of image reconstruction by VR-SGD algorithm is shown in Fig. 2. The first step is initialization where a DAS reconstructed image is applied as initial image $X_{0}$. The forward projection matrix is obtained from Eqs. (2) and (3). Other parameters are also initialized, including the tuning parameter λ, initial step size $\alpha_{0}$, decay constant a, the number of iterations for the outer loop N, and the batch size M that corresponds to the number of selected submatrix in each inner loop. The number of iterations for the inner loop is fixed to be the same as the number of channels Q. In the second step, the full gradient of the projection matrix A is computed for each outer loop. In the third step, a fixed number of submatrices $A_{i}$ are randomly selected and the gradients of the submatrices are computed for each inner loop. The updating direction d is the combination of the full gradient obtained from the full projection matrix A in the outer loop, and the gradient obtained from the submatrices $A_{i}$ in the inner loop. At the end of each inner loop, the image is updated with the computed direction multiplied by a proper step size $\alpha_{j}$. The update is realized by proximal gradient method, which accounts for the non-smooth TV term.^16^ In the following sections, the number of iterations refers to the iterations for the outer loop. Compared with conventional FGD algorithm that computes the full gradient of the cost function by dealing with a full projection matrix,^14^ VR-SGD takes less computational time because it does not need to update the full gradient in each inner loop. Meanwhile, by updating the full gradient in each outer loop, the convergence rate of VR-SGD is faster than that of SGD algorithm that randomly chooses a smaller size of submatrix of the projection matrix to compute the gradient.^15^

### 2.2 Numerical Simulation

**Fig. 3:**
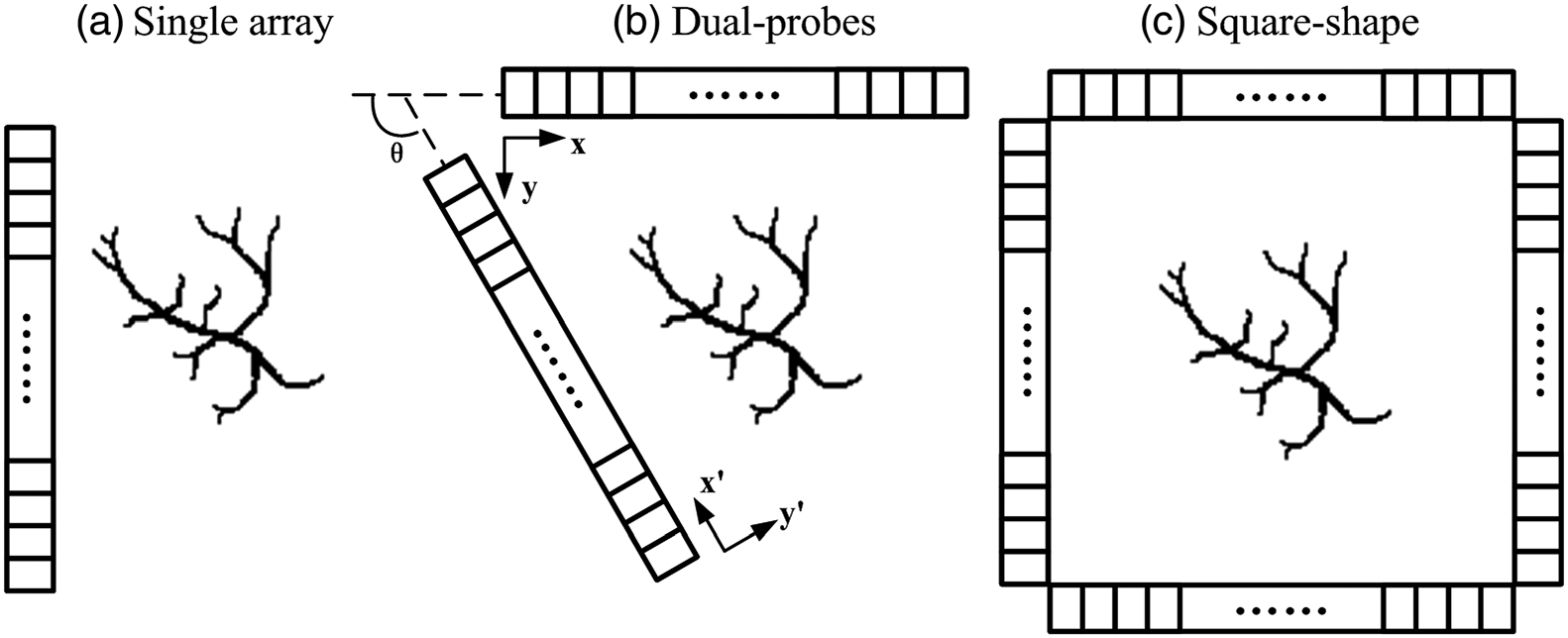
Detection geometries enabled by linear arrays: (a) single-array detection; (b) dual-probe geometry with an angle θ (e.g., parallel-shape has θ=180  deg and L-shape has θ=90  deg); and (c) square-shape geometry with four probes.

Numerical simulation is carried out to compare PAT reconstruction under various detection geometries enabled by linear arrays as shown in Fig. 3. The single-array geometry represents the traditional configuration where only one linear array is used, and all the transducer elements are located on one side. Dual-probe configuration is a potential approach for increasing the limited view, where two linear arrays form an angle of θ. As two special cases of dual-probe configuration, parallel-shape and L-shape geometries are investigated where θ=180  deg and θ=90  deg, respectively. To compare the image reconstruction with full-view detection, a square-shape geometry is also included, where four linear arrays fully enclose the region of the sample on four sides. In this study, each linear array contains the same number of 128 transducer elements.

Table 1 lists the main parameters used in the simulation. An object is defined in a thin slab with the physical dimension of 38  mm×38  mm, corresponding to 128×128  pixels. The linear transducer arrays are located inside the 2D plane of the object slab. The pitch size (0.3 mm) and total width (38 mm) of the transducer array are set to match with the dimensions of the linear array applied in the experiment. To synthesize the PA pressure wave p(t) generated from the object, 3D forward simulation by k-Wave toolbox is performed, where the pressure wave propagates in the entire 3D space extended from the object slab in the perpendicular direction while the receiving transducer arrays are located inside the 2D object slab. Image reconstruction is performed by DAS and VR-SGD in the 2D plane where the transducer elements are located. For VR-SGD reconstruction, the pressure wave p(t) is integrated to obtain the acoustic velocity potential $g(t)=t\int_{0}^{t}p(t^{\prime })dt^{\prime }$, and G is the matrix form of g(t). Image reconstruction is achieved by iteratively minimizing the cost function in Eq. 4 with the VR-SGD algorithm. During the iterations, G is used as the received PA data and the projection matrix A is used as the forward propagation model. The simulation is carried out in MATLAB R2018a on a laptop with 2.3 GHz Intel Core i5 CPU and 16 GB memory. The setting of the linear transducer array is referred to the one used in our experiment.

**Table 1:** Parameter settings for the simulation.

Parameter	Value
The physical size of the region	38 mm × 38 mm
Pixels	128 × 128
Speed of the sound	1500 m/s
Number of elements	128
Sampling rate	40 MHz
λ (TV tuning parameter)	$1\times 10^{-5}$
*M* (batch size)	5
*N* (number of outer loop iteration)	30
$\alpha_{0}$ (initial step size)	5
Decaying constant a	1.05

### 2.3 Experimental Design and Calibration

Experiments are also carried out to validate PAT reconstruction under a single-array and dual-probe detection geometries, as encouraged by the simulation results. The square-shape geometry is not studied experimentally given that it is less promising in practice. The details of the experimental setup can be found in our previous publication.^10^^,^^17^ The system includes a laser source, an optical parametric oscillator (OPO), a DAQ module, an US imaging system, and US linear array transducers. The laser is a Q-switched Nd:YAG laser (Surelite OPO Plus SLIII-10, Continuum, San Jose, United States). The laser wavelength can be tuned from 680 to 2500 nm by the OPO. The laser pulse width is 5 ns, and the repetition rate is 10 Hz. Two identical linear array transducers (L14-5/38, Analogic, Richmond, BC, Canada) are used and each has 128 elements with 7.2 MHz center frequency, minimum 70% fractional bandwidth (at −6  dB), and 0.3 mm element pitch. The transducers are connected to an US imaging system (SonixMDP, Analogic, Richmond, BC, Canada) through a DAQ module. The DAQ module acquires PA signals from 128 channels in parallel at a sampling rate of 40 MHz and 12-bit resolution. In the experiment, the signal G is obtained from the measured PA data. The batch size of VR-SGD is set to 10 to compute a more accurate gradient direction for the experimental data.

In the two-probe configuration, the two linear array transducers are manually positioned in the same imaging plane. Nevertheless, their relative angle and separation do not need to be known beforehand and the angle θ can be flexibly adjusted to fit the sample. In experiment, the relative positions of the two transducers are calibrated by transmitting an US signal from the first transducer while receiving the US signal with the second one.^10^ The coordinate system of the second transducer array is then calibrated and transformed into the coordinate system of the first array where the space of the reconstructed image is defined. The transformation can be described by two translational parameters h and k in the X and Y directions, respectively, and the rotational parameter θ. The global rigid transformation matrix can be written as

$$[\begin{array}{l} i\\ j\\ 1 \end{array}]={\left([\begin{array}{ccc} 1 & 0 & h\\ 0 & 1 & k\\ 0 & 0 & 1 \end{array}][\begin{array}{ccc} \cos\;\theta & -\sin\;\theta & 0\\ \sin\;\theta & \cos\;\theta & 0\\ 0 & 0 & 1 \end{array}]\right)}^{-1}[\begin{array}{l} i^{\prime }\\ j^{\prime }\\ 1 \end{array}],$$ (5)

where $\left(i^{\prime },j^{\prime }\right)$ denote the coordinate values of the second transducer in its own coordinate system, and (i,j) are the coordinate values of the second transducer transformed to the imaging space (defined in the coordinate system of the first transducer). In Eq. (5), the two transducers are assumed to be aligned in the same imaging plane, which is achieved by manual adjustment. The accuracy of this alignment is controlled by maximizing the received signal intensity during the US transmitting/receiving calibration process.^10^ In the VR-SGD reconstruction, the forward projection operator is applied on the combined data from both linear arrays.

### 2.4 Quantification Metrics

To compare the PAT reconstruction, different metrics are used based on whether the true data of the initial pressure is known or unknown. For simulation results where the initial pressure distribution is known, the root-mean-square error (RMSE) is computed. RMSE compares the pixel-value difference of two images, which is defined as

$$\mathrm{RMSE}=\sqrt{\frac{1}{mn}\overset{m}{\underset{i=1}{\sum }}\overset{n}{\underset{j=1}{\sum }}{\left[X(i,j)-Y(i,j)\right]}^{2}},$$ (6)

where X and Y denotes the reconstructed and the actual pressure distribution with m×n pixels, respectively.

For experimental results where the true value of the initial pressure distribution is not given, the contrast-to-noise ratio (CNR) and signal-to-noise ratio (SNR) are computed. Their definitions are

$$\mathrm{CNR}=20\;\log_{10}(\frac{\left|\mu_{i}-\mu_{o}\right|}{\sigma_{o}}),$$ (7)

$$\mathrm{SNR}=20\;\log_{10}(\frac{\mu_{i}}{\sigma_{o}}),$$ (8)

where $\mu_{i}$ is the mean signal intensity averaged over the pixels with intensity greater than 50% of the peak value inside a selected region of interest (ROI) of the target, $\mu_{o}$ is the mean background intensity averaged over a selected background region, and $\sigma_{o}$ is the standard deviation of the selected background region. The target and background ROIs are selected to be circular areas of the same size for each experimental image. In addition, generalized contrast-to-noise ratio (gCNR) is a recently developed metric that is calculated from the overlap of the probability density functions of ROIs inside the target and background regions. The gCNR value ranges from 0 to 1, which makes it robust against the variation of dynamic range when comparing different methods. In our study, gCNR is calculated for all the experimental results using the histogram-based method described in Ref. 18:

$$\mathrm{gCNR}=1-\overset{N}{\underset{k=1}{\sum }}\min(h_{i}(x_{k})h_{o}(x_{k})).$$ (9)

Here, $h_{i}$ and $h_{o}$ represent the normalized histograms of the target and background ROI, respectively, derived with N bins (where N=255 in our study) centered at $\left\{x_{0},x_{1},\ldots ,x_{N-1}\right\}$.

## 3 Results

### 3.1 Simulation Results

**Fig. 4:**
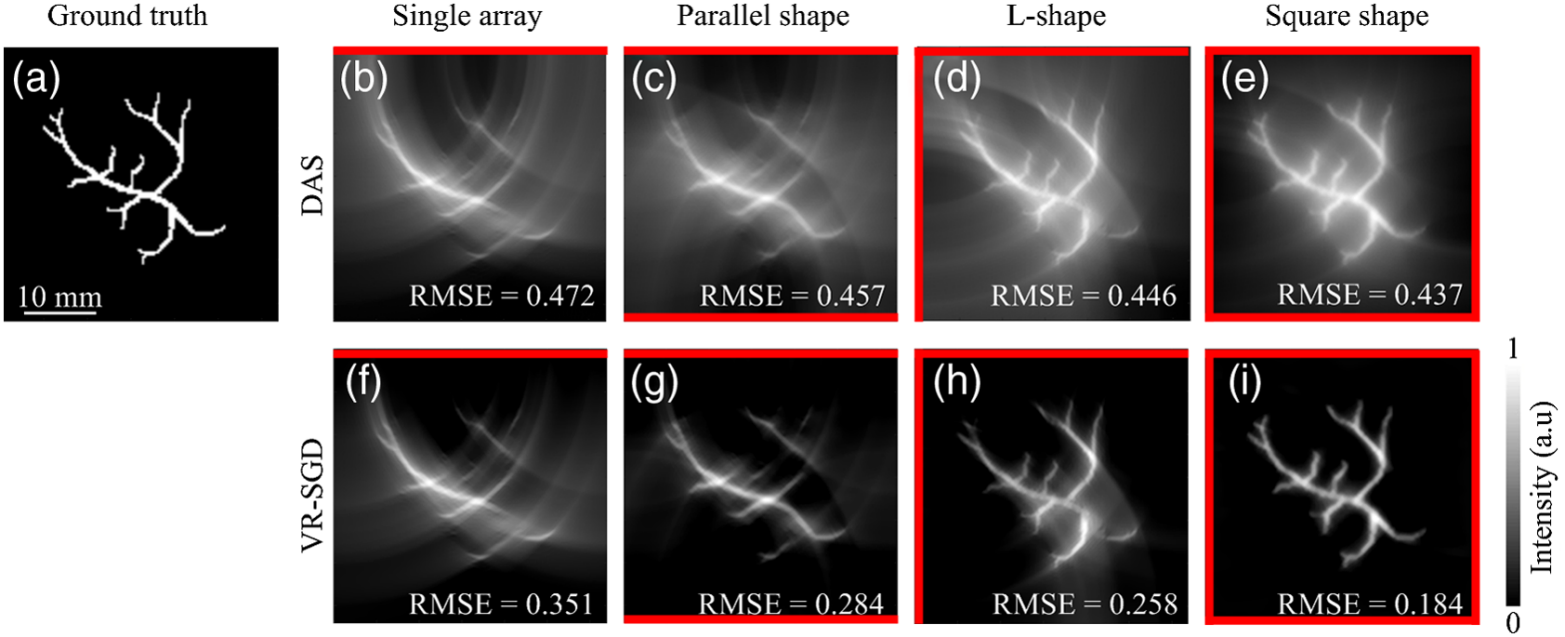
Reconstruction of the tree branch phantom by single-array, parallel shape, L-shape, and square shape detection geometries, respectively. (a) Initial pressure distribution of the phantom; (b)–(e) reconstructed images by DAS; and (f)–(i) reconstructed images by VR-SGD after 30 iterations. Positioning of the linear arrays is indicated by the red lines. RMSE value is labeled in each reconstructed image.

A simulation study is first carried out on a tree branch phantom that is frequently used to mimic blood vessels. Figure 4 shows the reconstruction results of the tree branch phantom by single-array, parallel shape, L-shape, and square shape detection geometries, respectively. The initial pressure distribution of the tree branch phantom is shown in Fig. 4(a). For each detection geometry, the DAS reconstructed image is shown in the top, the VR-SGD reconstructed image after 30 iterations in the bottom, and the orientation of the linear arrays are indicated by the red lines. The RMSE value is calculated and labeled in each reconstructed image.

For the single-array detection, strong arc-shaped artifacts are seen in the DAS reconstructed image due to the limited-view problem. With VR-SGD, the image reconstruction is improved with reduced artifacts and background noise. Nevertheless, as all the transducer elements are only located on the top side of the image, some features oriented perpendicularly to the transducer array are blurred or lost. Moreover, the intensity distribution of the tree branch is not homogeneous since features located on the lower half of the image are farther away from the transducer. For the parallel-shape dual-probe detection, more features are distinguishable than the result by single-array detection, and the intensity distribution looks more balanced. For the L-shape detection, most of the structures are reconstructed. Compared to the parallel-shape, L-shape geometry benefits from a broader view angle. In Fig. 4(h), with the L-shape detection and VR-SGD algorithm, almost all the artifacts caused by the limited-view issue have been removed except for the region located at the bottom right where the sample is farther away from the transducers in both directions. For the square-shape detection, although DAS can detect the complete structure, a relatively high-noise background still exists. Larger view angle and higher number of transducer elements lead to better results.^19^ With VR-SGD, the background noise is significantly reduced. The square-shape geometry provides a full-view condition where the reconstructed image represents the optimal quality. Although the square-shape configuration is not practical in clinical settings due to limited access to the tissue location, it is shown here as the optimal reference image to evaluate how close the other configurations can perform similarly as a full-view detection. For the single-array, parallel shape, L-shape, and square shape detection geometries, the RMSE is 0.472, 0.457, 0.446, and 0.437, respectively, with the DAS reconstruction. The relatively high RMSE is mainly due to the high background noise. Meanwhile, VR-SGD can significantly reduce the artifacts and noise background for each detection geometry. The RMSE are reduced to 0.351, 0.284, 0.258, and 0.184 in VR-SGD for the corresponding geometries, respectively.

**Fig. 5:**
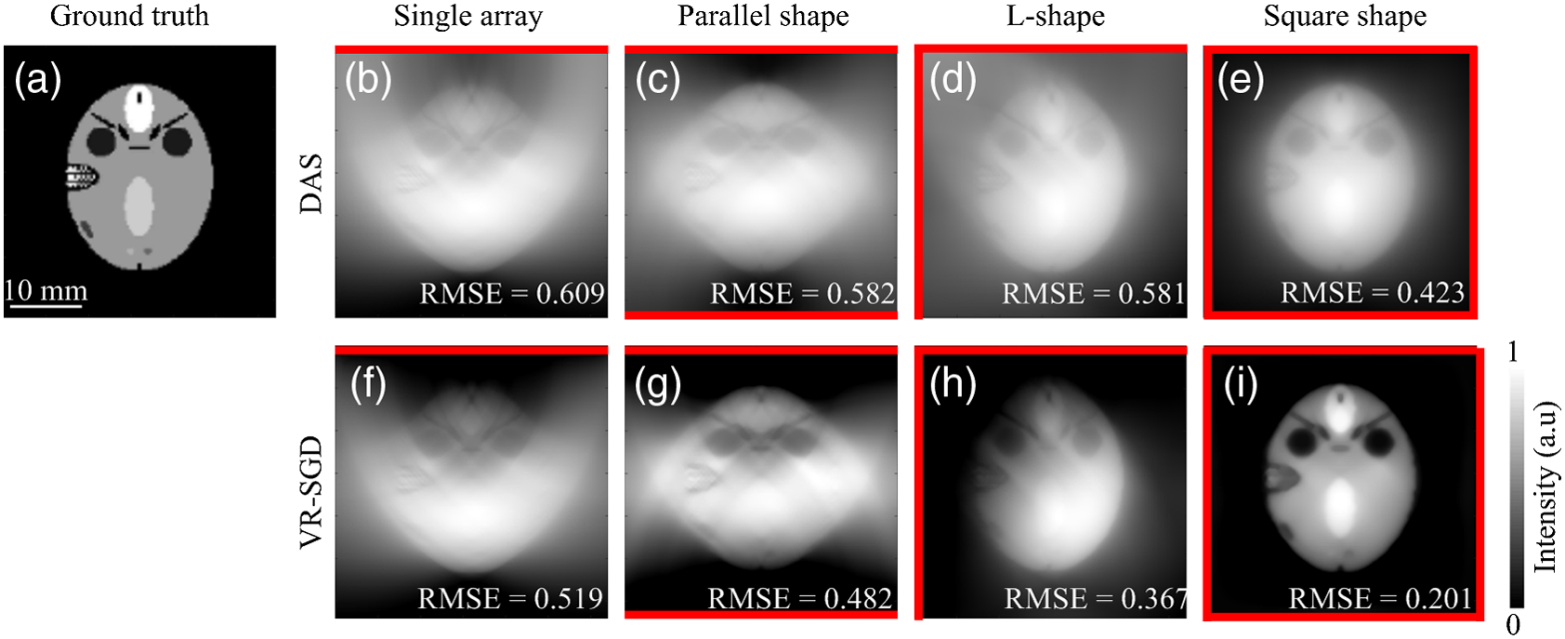
Reconstruction of the FORBILD phantom by single-array, parallel shape, L-shape, and square shape detection geometries, respectively. (a) Initial pressure distribution of the phantom; (b)–(e) reconstructed images by DAS; and (f)–(i) reconstructed images by VR-SGD after 30 iterations. Positioning of the linear arrays is indicated by the red lines. RMSE value is labeled in each reconstructed image.

A FORBILD phantom is also simulated as it provides more distinguishable features. Figure 5 shows the reconstruction results of the FORBILD phantom by single-array, parallel shape, L-shape, and square shape detection geometries, respectively. The initial pressure distribution of the FORBILD phantom is shown in Fig. 5(a). For each detection geometry, the DAS reconstructed image is shown in the top, the VR-SGD reconstructed image after 30 iterations in the bottom, and the orientation of the linear arrays are indicated by the red lines. The RMSE value is calculated and labeled in each reconstructed image. For the single-array detection, the DAS reconstructed image has extremely low quality with strong arc-shaped artifacts. VR-SGD improves the image by reducing the noise background. However, as the transducer elements are all located at the top side of the image, the structures that are perpendicular to the transducer array are blurred, which resulted in distortion and broadening of the vertical edges. For the parallel-shape dual-probe detection, the artifacts are reduced more than the single-probe detection. Nevertheless, the vertical edges still suffer from distortion and broadening. For the L-shape detection, the vertical edges can be reasonably detected with no obvious distortion or broadening. A clear contour profile with more visible structures is observed. The VR-SGD result shows much lower background and higher SNR than the DAS result. The L-shape geometry with VR-SGD performs reasonably well and can reconstruct most of the features. For the square-shape detection, complete structure of the FORBILD phantom is observed. For the single-array, parallel shape, L-shape, and square shape detection geometries, the RMSE is 0.609, 0.582, 0.581, and 0.423, respectively, with the DAS reconstruction. VR-SGD can significantly reduce the artifacts and noise background for each detection geometry. The RMSE are reduced to 0.519, 0.482, 0.367, and 0.201 in VR-SGD for the corresponding geometries, respectively.

**Fig. 6:**
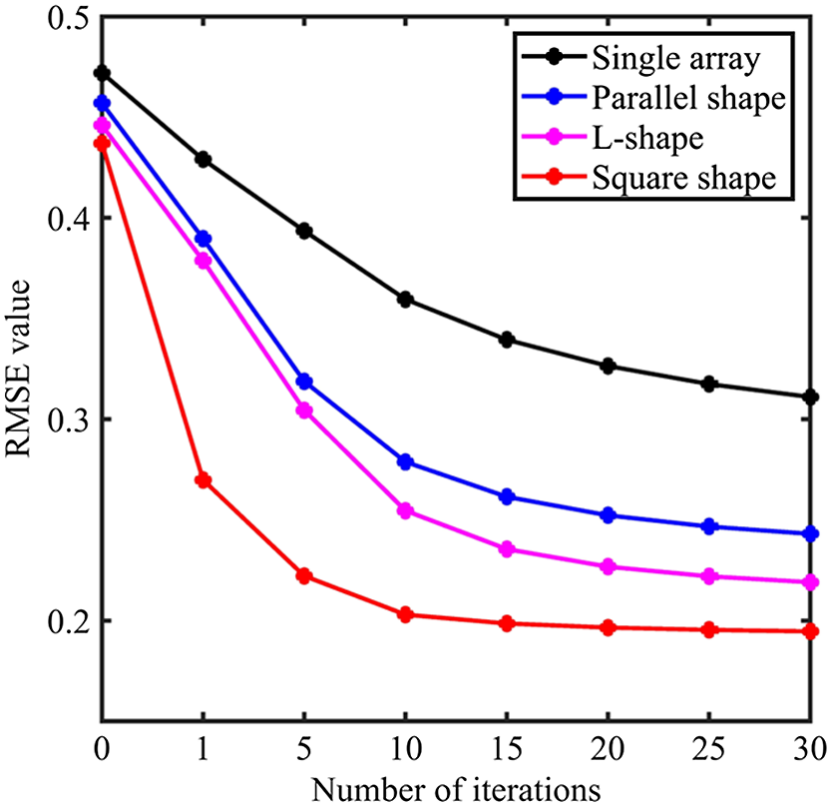
RMSE versus number of iterations in VR-SGD for different detection geometries on the tree branch phantom.

The VR-SGD algorithm optimizes the image reconstruction by iteratively reducing the cost function. Figure 6 shows the RMSE versus number of iterations in VR-SGD for the four detection geometries on the tree branch phantom. The square-shape and L-shape geometries show a faster convergence than the other two geometries. Moreover, the RMSE obtained by the L-shape geometry is close to that obtained by the square-shape geometry in terms of the saturated value and convergence rate. Therefore, the simulation demonstrates that dual-probe configuration with VR-SGD algorithm can significantly improve the PAT reconstruction in linear arrays with limited view. Especially, the L-shape geometry (θ=90  deg) performs comparably as well as that of the full-view detection by square-shape geometry.

In comparison to DAS, VR-SGD trades off the processing time to improve the image quality. The processing time of VR-SGD depends on the number of iterations and complexity of the gradient. Currently, it takes about 1.5 to 2.5 h for single-array detection, and 2 to 3 h for dual-probe detection to complete 30 iterations, respectively. On the other hand, DAS only takes about 1 min to reconstruct the image obtained either by single-array or dual-probe detection. The processing time can be reduced by upgrading the hardware and optimizing the software in the future. For example, GPU is powerful for graphic computation that can be implemented to enhance the processing speed.

### 3.2 Experimental Results

**Fig. 7:**
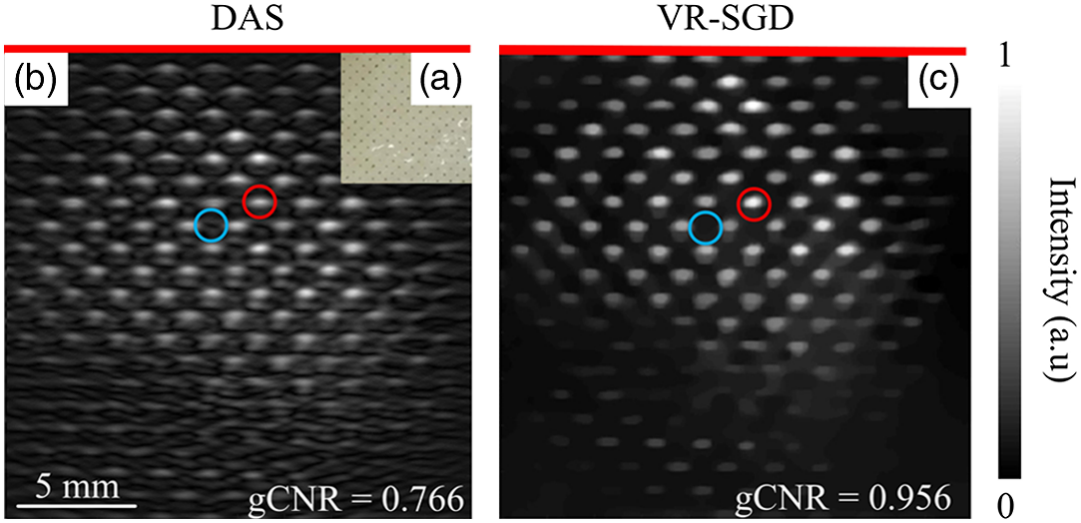
PAT imaging of printed dots array detected by single array. (a) Photograph of the phantom; (b) image reconstructed by DAS; and (c) image reconstructed by VR-SGD. The red line indicates the position of the transducer array. The ROIs for the signal and background are marked by the red and blue circles, respectively.

As guided by the simulation, PAT reconstruction is also validated with single-array and dual-probe detection geometries in experiments. Figure 7 shows the results detected by single array on a phantom of printed dots embedded in gelatin. A photograph of the phantom is shown in Fig. 7(a). The diameter of the dots is 0.1 mm and the gap between two neighboring rows or columns is around 1 mm. The laser illumination shines from the top of the sample plane. The linear array transducer is aligned in the sample plane and detects PA signals from one side of the phantom. The DAS reconstructed image is displayed in Fig. 7(b), where the dots show artifacts and elongation in the horizontal direction due to the limited-view problem. The VR-SGD reconstructed image is shown in Fig. 7(c), where the image shows reduced artifacts and cleaner background. Most of the dots are reconstructed and the dots show a smoother outline without noticeable artifacts. The background of the image is clearer, which leads to higher SNR. On the other hand, the TV regularization suppresses some information that has relatively low intensity and small size. The CNR and SNR for the image obtained by DAS are 16.2 and 18.9 dB, respectively. After applying VR-SGD, the CNR and SNR are enhanced to 36.1 and 37.5 dB, correspondingly. The gCNR is increased from 0.766 for DAS to 0.956 for VR-SGD.

**Fig. 8:**
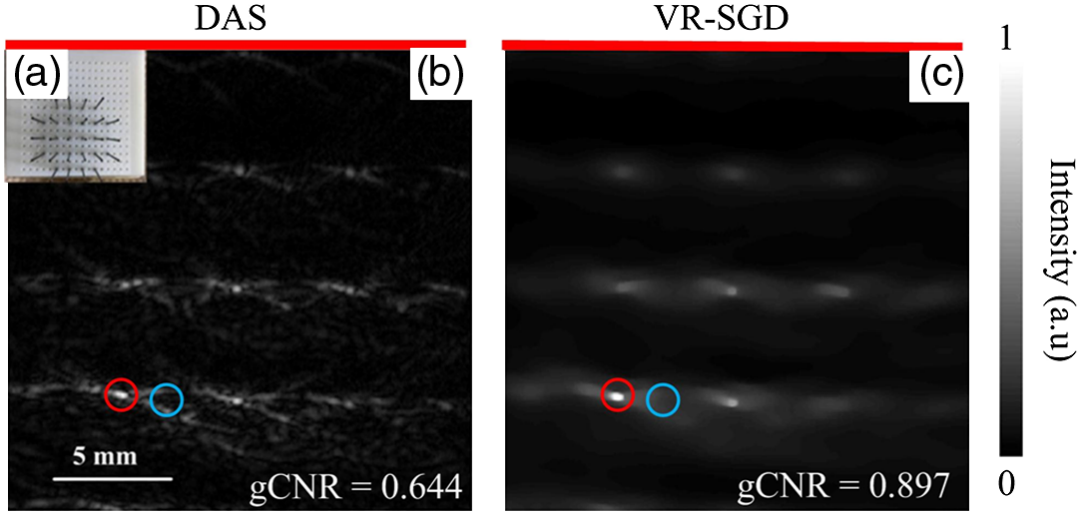
PAT imaging of pencil leads detected by single-array geometry: (a) photograph of the phantom; (b) image reconstructed by DAS; and (c) image reconstructed by VR-SGD. The red line indicates the position of the transducer array. The ROIs for the signal and background are marked by the red and blue circles, respectively.

Figure 8 shows PAT images of pencil leads by single-array detection. Figure 8(a) shows the photograph of the pencil leads, which are embedded in gelatin during imaging. The diameter of the pencil lead is 0.7 mm and the gap between two neighboring leads is 7 mm. Light illuminates the pencil leads from the side surface of the phantom, whereas the linear array receives the PA signal from the opposite side of the phantom. Although the PA signal is generated and propagates in 3D space, the linear array transducer mainly receives the in-plane signal. However, some out-of-plane signal can also be received by the transducer that can affect the image reconstruction. The DAS reconstructed image is shown in Fig. 8(b), which has strong artifacts. The VR-SGD reconstructed image is shown in Fig. 8(c). The image after iteration shows improved quality in reduced artifacts and cleaner background. Comparing DAS with VR-SGD, CNR is improved by 11.9 dB (from 11.0 to 22.9 dB), SNR is improved by 11.9 dB (from 14.9 to 26.8 dB), and gCNR is increased from 0.644 to 0.897. Nevertheless, the iteration could not remove all the artifacts, likely because of the out-of-plane signal.

**Fig. 9:**
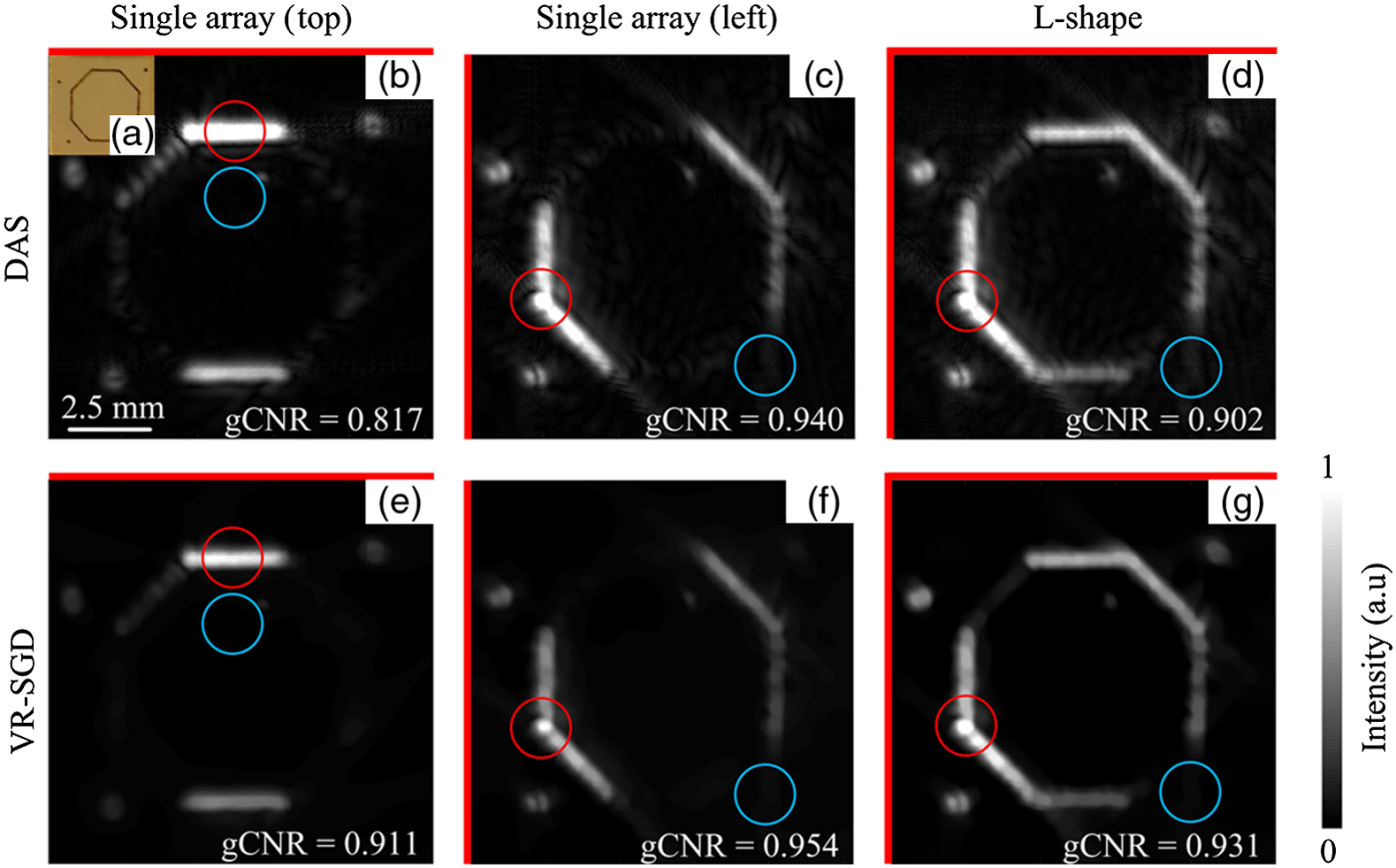
PAT images of octagon phantom detected by single-array and L-shape geometries. (a) Photograph of the phantom. (b)–(d) DAS reconstructed images by single array (top), single array (left), and L-shape detections, respectively. (e)–(g) VR-SGD reconstructed images for the corresponding detection geometries, respectively. Red lines indicate the orientation of the transducer array. The target and background ROIs are marked by the red and blue circles, respectively.

As it has been shown to be a promising detection geometry in simulation, the dual-probe configuration is also validated in experiment, where the angle θ is selected to be close to 90 deg (L-shape) for optimum reconstruction. Figure 9 shows the results obtained by single-array and dual-probe detections on a phantom with a printed octagon and three dots embedded in gelatin. Figure 9(a) shows a photograph of the phantom. The two transducers are placed on the top and left side of the object, where the actual angle is calibrated to be θ=110  deg. Figures 9(b)–9(d) show DAS reconstructed images by single array (top), single array (left), and L-shape detections, respectively. With single-array detection, certain structures are missed, mostly in the orientation perpendicular to the transducer array due to limited-view detection. Since DAS has no iteration, the L-shape detection result of Fig. 9(d) is obtained by simply adding the individually reconstructed images of Figs. 9(b) and 9(c). Even though Fig. 9(d) displays more complete structure, the artifacts and background noise have also been added. In comparison, Figs. 9(e)–9(g) show VR-SGD reconstructed images by single array (top), single array (left), and L-shape detections, respectively. In VR-SGD, the L-shape detection result of Fig. 9(g) is obtained by combining the PA data from the two probes and then run the iterations of optimization. The L-shape detection shows a more complete structure than single-array detection. VR-SGD reconstructed image shows reduced artifacts and noise background than the corresponding DAS results. For example, comparing the reconstructed image by DAS in Fig. 9(d) and by VR-SGD in Fig. 9(g), the CNR is increased by 4.2 dB (from 32.4 to 36.6 dB), SNR increased by 4.0 dB (from 32.8 to 36.8 dB), and gCNR increased from 0.902 to 0.931.

**Fig. 10:**
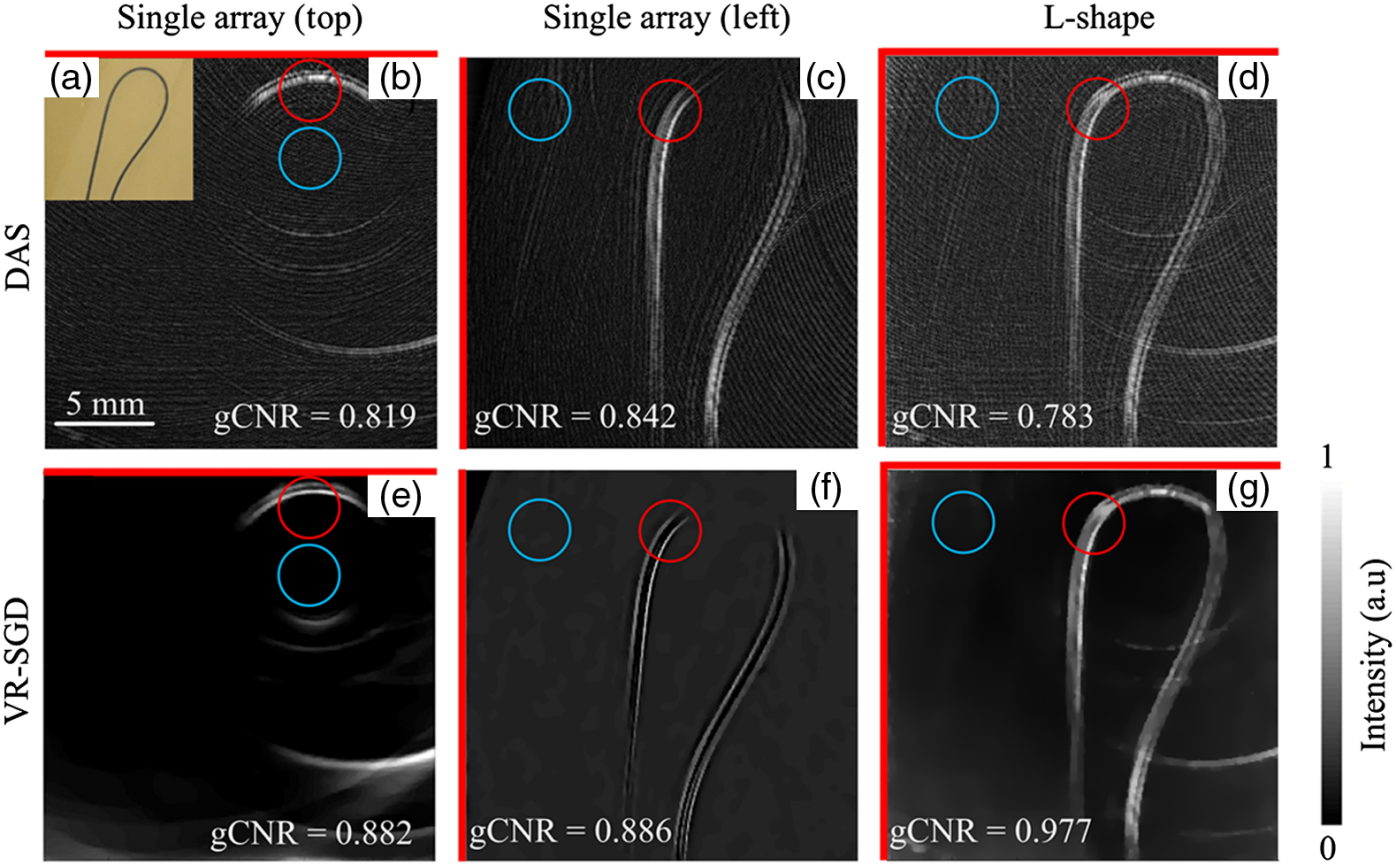
PAT images of a tube phantom detected by single-array and L-shape geometries. (a) Photograph of the phantom. (b)–(d) DAS reconstructed images by single array (top), single array (left), and L-shape detections, respectively. (e)–(g) VR-SGD reconstructed images for the corresponding detection geometries, respectively. Red lines indicate the orientation of the transducer array. The target and background ROIs are marked by the red and blue circles, respectively.

Figure 10 shows the experimental results from single-array and dual-probe detections on a PVC tube filled with methylene blue. Figure 10(a) shows a photograph of the phantom. The two transducers are placed on the top and left side of the object, where the actual angle is calibrated to be θ=89  deg. Figures 10(b)–10(d) show DAS reconstructed images by single array (top), single array (left), and L-shape detections, respectively. The result by single array only captures part of the structure that is parallel to the transducer array. With L-shape detection, a more complete structure is obtained on the expense of doubling the background noise. In comparison, Figs. 10(e)–10(g) show VR-SGD reconstructed images by single array (top), single array (left), and L-shape detections, respectively. The L-shape detection with VR-SGD algorithm shows a more complete structure and reduced background. The side wall of the tube can be distinguished. VR-SGD reconstructed image shows reduced artifacts and noise background than the corresponding DAS results. Comparing the reconstructed image by DAS in Fig. 10(d) and by VR-SGD in Fig. 10(g), the CNR is significantly increased by 30.2 dB (from 17.8 to 48 dB), SNR increased by 28.7 dB (from 21.1 to 49.8 dB), and gCNR increased from 0.783 to 0.977.

As gCNR only compares the intensities of the target and background ROI, respectively, it is sensitive to where the ROIs are selected and the background noise. The image by the L-shape detection not only combines the structures from each probe but may also add up the background noise. The precision of the alignment and calibration of the two coordinate systems of the two probes can also affect the gCNR. Therefore, the gCNR of the images from single array could be slightly higher than that from the L-shape as seen in Figs. 9 and 10. Nevertheless, the main advantage of applying dual-probe detection is to increase the view angle, which can then lead to a more complete image reconstruction in terms of structures. In Figs. 9 and 10, it is clear that a more complete structure (less missing structure) is obtained with the L-shape detection than with a single array. A limitation of gCNR (as well as CNR and SNR) is that it does not represent the similarity between the reconstructed image and the original object. Although quantification of similarity can be achieved by RMSE, it may be difficult to quantify such similarity in experimental study when ground truth is normally unknown.

The quantitative comparison of image reconstruction by DAS and VR-SGD is summarized in Table 2 for the experimental results. Image reconstructed by VR-SGD shows significant improvement than that by DAS. The amount of improvement varies and depends on the detection geometry and sample property.

**Table 2:** Quantitative comparison of PAT reconstruction by DAS and VR-SGD.

Detection	Sample	CNR (dB)		SNR (dB)		gCNR	
		DAS	VR-SGD	DAS	VR-SGD	DAS	VR-SGD
Single-probe	Dots array (Fig. 7)	16.2	36.1	18.9	37.5	0.766	0.956
	Pencil leads (Fig. 8)	11.0	22.9	14.9	26.8	0.644	0.897
Dual-probe	Octagon phantom [Fig. 9(d) versus Fig. 9(g)]	32.4	36.6	32.8	36.8	0.902	0.931
	Tube phantom [Fig. 10(d) versus Fig. 10(g)]	17.8	48	21.1	49.8	0.783	0.977

## 4 Discussion

As linear array transducers are widely used in clinical US imaging, PAT with linear array is highly approachable and feasible for clinical applications. However, due to the limited-view problem, the single-array geometry has limitations in its performance, which has hindered its broader applications. To explore the potential of linear transducers and to address the limited-view problem, various detection geometries enabled by linear transducers are investigated. Although a square-shape geometry can provide full view, it is very demanding on the hardware requirement and less feasible to access the organ from four sides in clinical applications. On the other hand, the dual-probe detection geometry is shown to be highly efficient in addressing the limited-view problem and is quite feasible in clinical applications. With our calibration approach, the dual probes can be positioned flexibly with an arbitrary angle θ to fit the organ. For the detection with a single-linear array, certain structures that are oriented perpendicularly to the probe may not be detected because the PA wave forms a plane wave propagating perpendicularly to the probe direction. With the L-shape detection of θ=90  deg, a second probe can capture those missing structures. With the parallel-shape detection of θ=180  deg, it can improve the uniformity of the signal intensity especially on the far side of the first single-linear probe. The actual angle θ that can be implemented in practice will depend on the geometry and accessibility of the imaging site.

Recently, Nyayapathi et al.^20^ reported a dual-probe PAT system for *in vivo* imaging of human breast. The two probes are placed in a parallel-shape configuration, with detection from the top and bottom sides of the breast simultaneously. They obtained a 7-cm penetration depth that could cover the entire breast with slight compression. Breast imaging is an application area where the dual-probe detection can be implemented. Although the parallel-shape geometry is already shown to enhance the penetration depth, the L-shape configuration could also be implemented to further improve the limited-view problem. Prostate imaging is another application area where dual-probe detection is possible. In simulation, Moradi et al.^21^ demonstrated two configurations for prostate imaging, by a transrectal ultrasound transducer (TRUS) from the posterior side and a pick-up transducer from the anterior abdomen side. Thus it is possible to use a TRUS transducer and a pick-up transducer simultaneously to perform dual-probe PAT imaging to improve the image reconstruction. For prostate imaging, the anatomy of the body may limit the angle to be θ≈180  deg, which should still outperform a signal array.

Our study also shows that iterative algorithm, such as VR-SGD, can further address the limited-view problem by iteratively minimizing a cost function. With an accurate model of the forward projection matrix of the PA signal, VR-SGD can recover the missing structures and reduce the artifacts caused by limited-view problem. Currently, the projection matrix in Eq. 2 is a simplified model of PA signal propagation. In the current model, the directivity pattern of the transducer is included as a weighting factor D(i,k) based on the angle subtended from the pixel to the transducer element. In our experiment, this angle is relatively small because the sample is usually placed 30 to 50 mm away from the linear transducer. For example, for a typical ROI of $15\times 15\;{\mathrm{mm}}^{2}$, for the transducer element in the center, the angle is estimated to be 0.24 radians, where the sensitivity only slightly drops to 95% (−0.4  dB) for our transducer.^22^ The maximum angle occurs for the end element and is estimated to be 0.72 radians, where the sensitivity drops to 40% (−8  dB).^22^ By comparing the reconstructed images with and without directivity pattern, the effect of the directivity pattern was found to be not significant in our current study. The forward projection model can be further improved in the future by considering other factors such as the attenuation of light and the limited bandwidth of the transducer. In our previous study,^23^ a compensation approach has been implemented to counteract the effect of light attenuation when imaging deep into tissue. The consideration of limited bandwidth of the transducer has been reported by Shang et al.,^13^ where a convolution of the pressure wave with the impulse response is applied during iteration. With all those factors included, a more accurate projection matrix in 3D can be developed in the future.

Future work can also address a more comprehensive study of the effect of other iterative methods on the reconstruction with different transducer configurations. For example, the two-step iterative shrinkage/thresholding (TwIST) algorithm consists of two steps of iterative shrinkage/thresholding structure where each iteration depends on the previous two iterations rather than only the previous one.^13^ For many different regularizers, TwIST shows a faster convergence rate. On the other hand, the alternating direction method of multipliers (ADMM) algorithm exhibits a linear convergence rate as data is processed in parallel cores.^24^ Compared to the gradient descent methods, ADMM avoids gradient vanishing problems as it does not need gradient steps. Recently, deep learning-based methods were also applied to PA image reconstruction obtained by a linear array transducer.^25^^,^^26^ Johnstonbaugh et al.^25^ introduced a convolution neural network to PA imaging by linear array detection to locate deep samples. Vu et al.^26^ used a Wasserstein generative adversarial network to reduce the limited-view and limited-bandwidth artifacts. Although those methods mainly use simulated PA data for training, getting experimental data for training may be necessary in the future to represent more realistic situations.

## 5 Conclusion

The image reconstruction of PAT system using linear array transducers have been investigated, which is commonly affected by the limited-view problem. Different detection geometries enabled by linear arrays have been compared and the VR-SGD iterative algorithm has also been applied to PAT image reconstruction. The quality of the reconstructed image by dual-probe geometry is superior to those obtained by a single array and comparable to those obtained by full-view geometry in terms of the RMSE value. In experiments, PAT imaging with a single-probe and dual-probe detections is also validated, where the dual-probe detection can capture more complete structures. In addition, VR-SGD algorithm further enhances the reconstruction than DAS with improved CNR, SNR, and gCNR. Therefore, PAT with dual-probe detection and iterative reconstruction is shown to be a potential solution against the limited-view problem of linear arrays in clinical applications.
